# Diagnostic value of plasma RNF180 gene methylation for gastric cancer: A systematic review and meta-analysis

**DOI:** 10.3389/fonc.2022.1095101

**Published:** 2023-01-10

**Authors:** Tongxin Wang, Yating Zhang, Jianrong Wang, Yumin Li

**Affiliations:** ^1^ The Second Clinical Medical College, Lanzhou University, Lanzhou, China; ^2^ Gansu Provincial Key Laboratory of Gastrointestinal Tumor, Lanzhou University, Lanzhou, China

**Keywords:** RNF180, gastric cancer, methylation, diagnosis, meta - analysis

## Abstract

**Objective:**

A systematic evaluation of the diagnostic value of Ring finger protein 180 (RNF180) gene methylation as a novel tumor marker for gastric cancer (GC) is required to improve the early diagnosis of gastric cancer patients.

**Methods:**

Computer searches of PubMed, Web of Science, Embase, The Cochrane Library, CNKI, CBM, WanFang Data, National Research Register, Cclinical Controlled Trials, Opengrey and VIP databases were conducted from the database’s inception to September 1, 2022. Two researchers independently screened the literature, extracted information, and assessed the risk of bias in studies that were included. The meta-analysis was carried out using RevMan 5.3 and Stata 16.0 software.

**Results:**

A total of 9 studies with a total of 1531 subjects were included. A random-effects meta-analysis revealed that the combined sensitivity (SEN), specificity (SPE), positive likelihood ratio (PLR), negative likelihood ratio (NLR), and diagnostic odds ratio (DOR) of plasma RNF180 gene methylation for the diagnosis of GC were: 0.54 [95% CI (0.45, 0.62)], 0.80 [95% CI (0.72, 0.87)], 2.73 [95% CI (2.09, 3.57)], 0.58 [95% CI (0.51, 0.65)], 4.74 [95% CI (3.59, 6.62)], respectively.

**Conclusion:**

The detection of RNF180 gene methylation in plasma has a high diagnostic value for GC and is expected to be a potential biomarker for the diagnosis of gastric cancer, according to current evidence.

**Systematic review registration:**

https://www.crd.york.ac.uk/PROSPERO/display_record.php?RecordID=370903, identifier CRD42022370903.

## 1 Introduction

Gastric cancer (GC) is a common type of digestive system malignant tumor. In 2020, the number of new cases surpassed 1 million, and the number of deaths surpassed 760,000, placing it fifth and fourth among global cancers in terms of incidence and mortality rates, respectively (1). Due to the lack of obvious clinical manifestations in the early stage, most gastric cancer patients are diagnosed at an advanced stage and miss the best time for surgical treatment, and have a poor prognosis (2). Therefore, the key to raising the survival rate and improving the prognosis of stomach cancer patients is early screening and detection. Currently, endoscopy is the most reliable screening and diagnostic tool, and gastroscopy in conjunction with a histopathological examination is the gold standard for detecting stomach cancer. However, because of the pain of the patient and problems like bleeding and perforation during the inspection, its clinical application is currently limited (3). Hematologic tumor markers, in contrast, have broad application potential, strong practicability, are less invasive, and offer significant economic benefits. Carcinoembryonic antigen (CEA), carbohydrate antigen 199 (CA199), carbohydrate antigen 724 (CA724), alpha-fetoprotein (AFP), and carbohydrate antigen 125 are currently accessible tumor markers for the screening or early identification of gastric cancer. However, the tumor markers have low sensitivity and specificity, which limits their utility in diagnosing gastric cancer (4, 5). Therefore, finding new and more effective tumor markers is essential for making an early diagnosis of stomach cancer.

DNA methylation is an epigenetic mechanism that occurs through the covalent addition of methyl groups to DNA (6). DNA methylation alters the conformation of genes, preventing them from binding to transcription factors and instead binding to methylated CPG-binding domain proteins. The latter in turn recruits other proteins and eventually forms dense and inactive abnormal chromatin, which can inactivate tumor suppressor genes and promote cancer progression (7, 8). One potential biomarker for the early identification of cancer is the methylation of the cancer-related gene (9).

Ring finger protein 180 (RNF180) is a tumor suppressor gene located on the long arm of chromosome 5. RINES is an E3 ring finger protein with ubiquitin ligase activity, produced by the gene encoding RNF180 (10). By mediating protein degradation *via* the ubiquitin-proteasome system (UPS), RNF180 participates in several biological processes including apoptosis, gene transcription, and DNA repair (2, 11, 12). When RNF180 was methylated, it led to structural and functional alterations in the gene that made it less effective at promoting apoptosis and failed to stop the growth, differentiation, and metastasis of the tumor (2, 13–17). Recent clinical studies have demonstrated a strong correlation between the prognosis, overall postoperative survival, lymph node metastases in patients with gastric cancer, and the RNF180 gene methylation status (18–21). RNF180 methylation was demonstrated by Cheung et al. to be detectable in plasma samples from 56.25 (18/32) gastric cancer patients, but not in plasma from 64 healthy controls (11). Similar results were discovered in the Zhang et al. investigation. According to their findings, RNF180 methylation was found in 57.89% (33/57) of plasma samples from patients with gastric cancer, but only in 23.81% (10/42) of the people in the control group (22). Accordingly, it can be inferred that RNF180 methylation may be an important marker for the diagnosis of gastric cancer.

There is a paucity of evidence-based medical evidence about the diagnostic effectiveness of RNF180 gene methylation in the diagnosis of gastric cancer. To establish a reference basis for early clinical screening of gastric cancer, this study aims to comprehensively compile the current studies investigating the relationship between RNF180 gene methylation and gastric cancer diagnosis. This will allow for a systematic evaluation of the feasibility of the plasma RNF180 gene methylation test as a diagnostic biomarker for gastric cancer.

## 2 Materials and methods

The Preferred Reporting Items for Systematic Reviews and Meta-Analyses on Diagnostic Test Accuracy (PRISMA-DTA) were used to conduct this systematic review and meta-analysis. Our study has been registered with PROSPERO (registration number: CRD42022370903).

### 2.1 Inclusion and exclusion criteria

The following were the inclusion criteria: 1) Diagnostic accuracy studies. 2) Patients diagnosed with GC by the gold standard, regardless of age, gender or race. 3) Studies analyzing the relationship between RNF180 gene methylation status and gastric cancer. 4) Studies providing sensitivity (SEN), specificity (SPE), positive likelihood ratio (PLR), negative likelihood ratio (NLR), diagnostic odds ratio (DOR), and area under the summary receiver operating characteristic curve (SROC) (AUC).

The following were the exclusion criteria: 1) Repeatedly published research. 2) Unable to obtain the full text or extract the data of the four-cell **Table 3**) Reviews, case reports, and conference abstracts.

### 2.2 Data sources and searches

Computer searches of PubMed, Embase, The Cochrane Library, Web of Science, CNKI, WanFang Data, VIP, National Research Register, Clinical Controlled Trials, Opengrey and CBM databases were searched to collect clinical studies on plasma RNF180 gene methylation for the diagnosis of gastric cancer. The search time frames ranged from the database’s inception to September 1, 2022. In addition, to supplement access to relevant literature, a combination of Google Scholar and references from the included literature was used. The searches used a combination of subject terms and free terms. Using PubMed as an example, the search strategy is as follows: (Stomach Neoplasms OR gastric cancer OR gastric carcinoma OR stomach cancer OR Stomach Neoplasm OR stomach carcinoma OR gastric adenocarcinoma) OR ((gastric OR stomach) AND (cancer OR carcinoma OR tumor OR malignancy OR neoplasm OR adenocarcinoma))) AND (rnf180 OR rines OR ring finger protein 180)) AND (methylation).

### 2.3 Literature screening and data extraction

Two researchers independently screened the literature, and extracted and cross-checked the data based on the inclusion/exclusion criteria. Disagreements were settled through discussion or consultation with a third party. The title and abstract of the text were read first, and after excluding irrelevant literature, the full text was read again to determine inclusion. If information is missing from the study and data cannot be extracted, we contact the author by email to obtain the relevant information based on the email address of the corresponding author indicated in the included literature. The following data were extracted: 1) basic characteristics of the study: first author, year of publication, country, study type, sample size, and methylation detection method; 2) subject characteristics: gender, age, race, the amount of RNF180 methylation occurring in case and control groups, and source of the control group; 3) diagnostic information: sensitivity, specificity, and area under the curve (AUC).

### 2.4 Methodological quality assessment

Two investigators independently assessed the risk of bias in the included studies, and the results were cross-checked. The Quality Assessment of Diagnostic Accuracy Studies-2 (QUADAS-2) tool was used to assess the risk of bias (23). Each bias risk item was graded as “yes,” “no,” or “unclear,” while applicability concerns were graded as “high,” “low,” or “unclear.”

### 2.5 Statistical analysis

The combined sensitivity (SEN), specificity (SPE), positive likelihood ratio (PLR), negative likelihood ratio (NLR), and diagnostic odds ratio (DOR) were calculated using RevMan 5.3 and Stata 16.0 software, and the hierarchical summary receive operating characteristic was plotted (HSROC). The Fagan nomogram was used to calculate the positive post-test probability (PPP) and negative post-test probability (NPP). The χ2 test (α=0.05) was used to assess heterogeneity between the results of each study, and the magnitude of heterogeneity was quantified by combining I^2^. If there was no significant heterogeneity among the results, Meta-analysis was performed using a fixed-effects model. If the study results were heterogeneous, the sources of heterogeneity were investigated further using meta-regression and subgroup analysis. After excluding the effects of significant heterogeneity, Meta-analysis was performed using a random-effects model. Publication bias between the included studies was evaluated by drawing Deek’s funnel plot (with P > 0.05 indicating no publication bias).

## 3 Results

### 3.1 Search results

**Figure 1:**
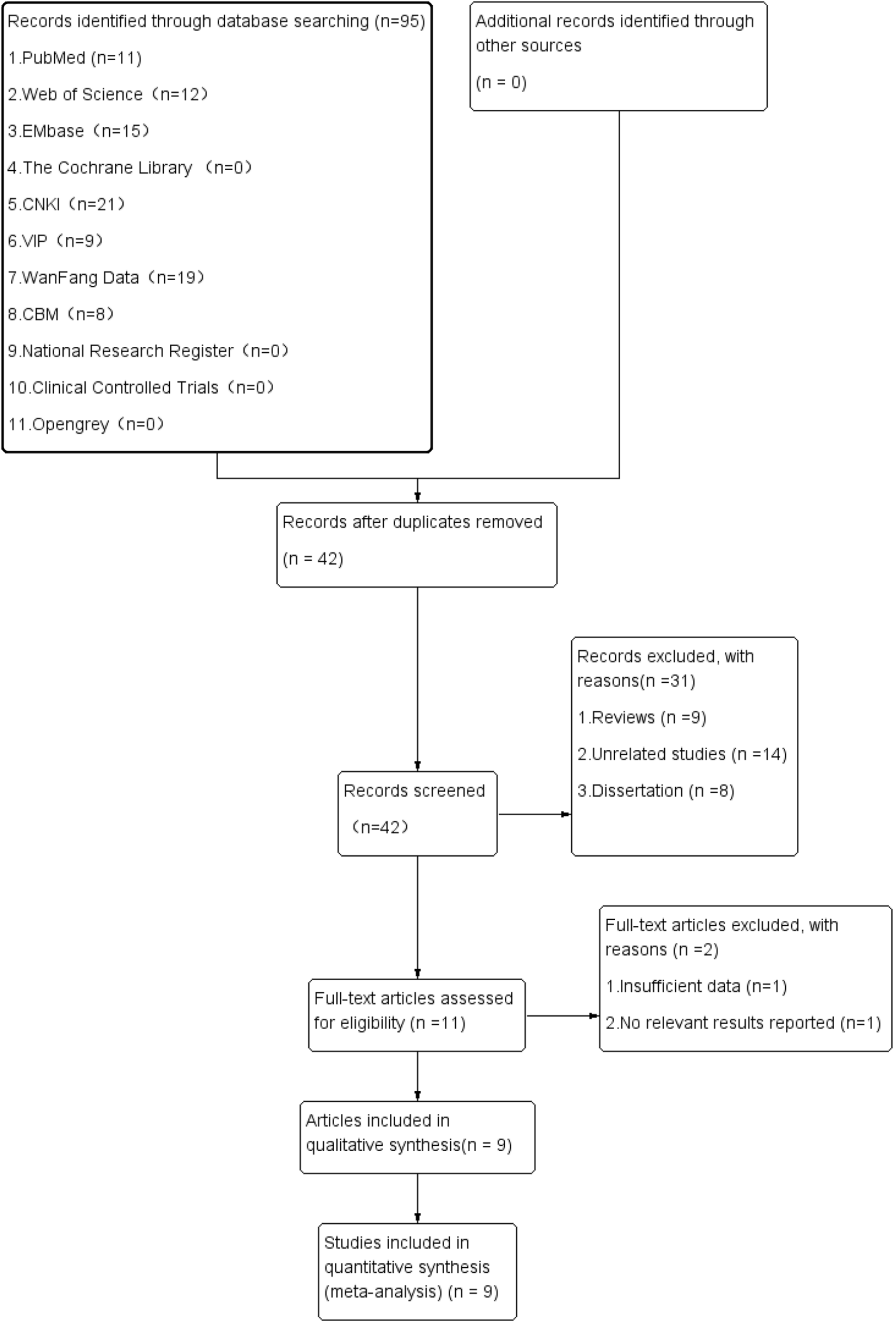
Literature screening process and results.

For the initial review, 95 relevant pieces of literature were obtained. Following the layer-by-layer screening, 9 studies on diagnostic accuracy with 1531 subjects were finally included (2, 11, 22, 24–29). **Figure 1** depicts the screening process and results of the literature.

### 3.2 Study characteristics

**Table 1** shows the basic characteristics of the included studies. The number of gastric cancer patients included in the study ranged from 32 to 151. All gastric cancer patients were diagnosed using the gold standard of gastroscopy combined with histopathology, and no radiotherapy or chemotherapy had been administered before surgery.

**Table 1:** Main characteristics of 9 studies included in the meta-analysis.

First author	Publication year	Country	Size	case		Control		Technology	Sensitivity	Specificity	Study design	Source of the control group
				M+	M−	M+	M−					
Zhao, L (24)	2022	China	156	43	17	39	57	MSP^1^	0.71	0.59	Retrospective	CSG^2^, CAG^3^, GU^4^
Zhang, M (25)	2022	China	80	35	20	7	18	MSP	0.63	0.72	Retrospective	healthy volunteers
Tan, Z. J (26)	2021	China	160	69	41	15	35	MSP	0.63	0.70	Retrospective	healthy volunteers
Xu, J. B (27)	2021	China	518	56	95	42	325	MSP	0.37	0.88	prospective	GID^5^, noncancer GID, NED^6^
Cao, C. Q (2).	2020	China	221	24	50	15	132	MSP	0.32	0.90	prospective	BGD^7^, NED
Song, Y (28)	2015	China	118	36	31	12	39	MSP	0.54	0.77	Retrospective	healthy volunteers
Zhang, X (22)	2014	China	99	33	24	10	32	MSP	0.58	0.76	Retrospective	healthy volunteers
Zhang, X.S (29)	2014	China	83	32	19	7	25	MSP	0.63	0.78	Retrospective	healthy volunteers
Kin‐Fai Cheung (11)	2012	China	96	18	14	0	64	qMSP^8^	0.56	1	Retrospective	healthy volunteers

^1^MSP, methylation-specific polymerase chain reaction.

^2^CSG, chronic superficial gastritis.

^3^CAG, chronic atrophic gastritis.

^4^GU, gastric ulcer.

^5^GID, gastrointestinal disease.

^6^NED, no evidence of disease.

^7^BGD, benign gastric diseases.

^8^qMSP, quantitative methylation-specific polymerase chain reaction.

### 3.3 Risk of bias and applicability concerns within studies

**Figure 2:**
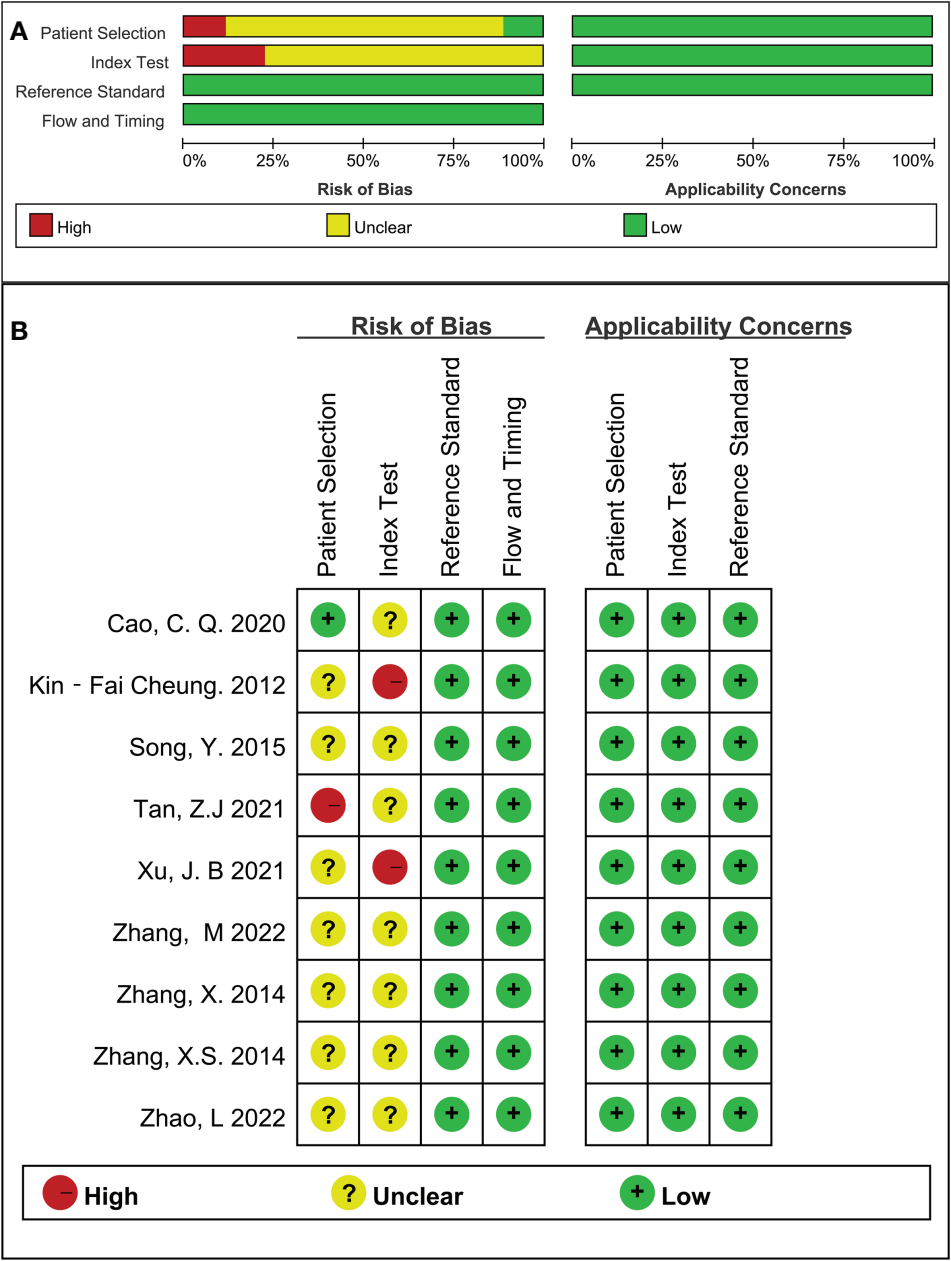
Quality assessment results **(A)**: Summary of methodological quality; **(B)**: Methodological quality map.

None of the included studies met all domain criteria of the QUADAS-2 methodological quality tool. On average, each study met two of the four risks of bias domains. The case-control design and inappropriate exclusions (for specific diagnoses) explain why almost no studies had low risk in the domains of patient selection and index testing. One study was rated as high risk in the area of patient selection because the exact time frame and continuity were not mentioned. All articles met the applicability criteria for all three domains of concern (**Figure 2**).

### 3.4 Meta-analysis

**Figure 3:**
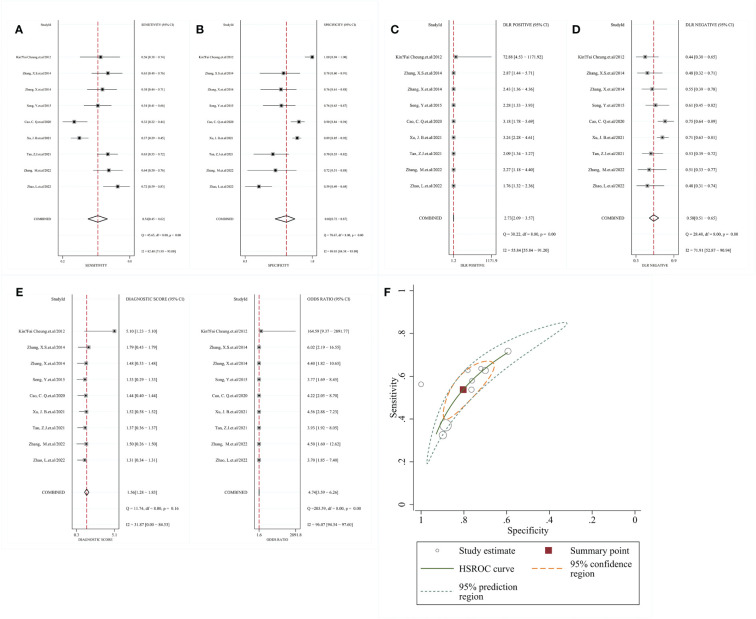
Meta-analysis results of RNF180 methylation for the diagnosis of gastric cancer **(A)** sensitivity; **(B)** specificity; **(C)** PLR; **(D)** NLR; **(E)** DOR; **(F)** HSROC; PLR, positive likelihood ratio; NLR, negative likelihood ratio; DOR, diagnostic odds. ratio; HSROC, hierarchical summary receives operating characteristic.

The included studies showed relatively high heterogeneity in terms of sensitivity and specificity (I²=82.48 and I²=89.83) using a random effects model, and Meta-analysis showed that SEN=0. 54 [95% CI (0.45, 0.62)], SPE=0.80 [95% CI (0.72, 0.87)], PLR=2.73 [95% CI (2.09, 3.57)], NLR=0.58 [95% CI (0.51, 0.65)], DOR=4.74 [95% CI (3.59, 6.62)] (**Figure 3**). The HSROC model gave a β estimate with a 95% confidence interval of 0.31 (-0.16, 0.78) and a Z-statistic of 1.30, corresponding to P=0.194. The Lambda estimate with a 95% confidence interval was 1.38 (1.01, 1.75) (**Figure 3F**).

**Figure 4:**
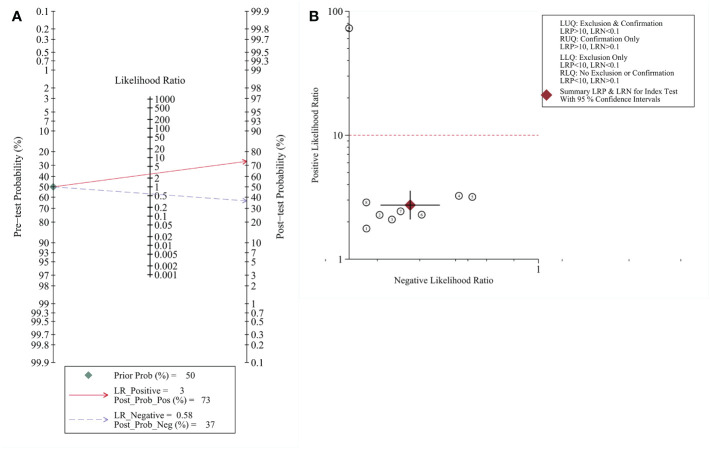
**(A)** Fagan nomogram plot; **(B)** Likelihood ratio coordinate plot.

When defining a pretest probability of 0.50 for RNF180 methylation to diagnose gastric cancer, the PPP and NPP were 73% and 37%, respectively (**Figure 4A**). The likelihood ratio dot plot of RNF180 gene methylation for the diagnosis of gastric cancer is shown in **Figure 4B**.

Thus, the findings suggest that the methylation status of the RNF180 gene has good diagnostic accuracy for gastric cancer and can be used as an effective biomarker for the disease.

### 3.5 Meta-regression and subgroup analysis

To investigate the sources of heterogeneity, we performed a meta-regression analysis based on the study design, methods of detecting methylation, sample size, and source of the control group (**Table 2**). The four covariates had no significant effect on the study’s diagnostic accuracy (P>0.05), according to meta-regression analysis. **Table 3** shows subgroup analyses to determine the source of the heterogeneity.

**Table 2:** Potential sources of heterogeneity detected by Meta-Regression.

Study covariates	Coefficient	standard error	*P*-value	RDOR	95%CI
study design^1^	0.39	2.01	0.86	1.48	(0.00;881.95)
methods of detecting methylation^2^	3.77	2.83	0.28	43.20	(0.01;350830.05)
sample size^3^	-0.24	0.401	0.60	0.79	(0.22;2.79)
source of the control group^4^	0.12	0.86	0.90	1.12	(0.07;16.90)

^1^The study design was divided into a retrospective study and a prospective study.

^2^The methods for detecting methylation were divided into MSP and qMSP.

^3^The sample size was divided into ≥100 subgroups and <100 subgroups.

^4^The source control group was divided into a healthy group and a Non-cancerous patient group.

**Table 3:** Results of subgroup analysis of RNF180 gene methylation were reported from nine studies in a diagnostic meta-analysis.

Analysis	Subgroup	No. of studies	Sensitivity (95% CI)	Specificity (95% CI)	PLR (95% CI)	NLR (95% CI)	DOR (95% CI)	AUC (95%CI)	I² (%)	p-value
Overall		9	0.54(0.45,0.62)	0.80(0.72,0.87)	2.73(2.09,3.57)	0.58(0.51,0.65)	4.74(3.59,6.62)	0.71(0.67,0.75)	0	0.535
Sample size	≥100	5	0.61(0.53;0.67)	0.85(0.79;0.90)	2.38(1.81;3.14)	0.65(0.56;0.75)	4.14(3.11;5.52)	0.7097	0	0.987
	<100	4	0.49(0.45;0.54)	0.83(0.80;0.85)	2.38(1.81;3.14)	0.65(0.56;0.75)	4.14(3.11;5.52)	0.7097	48.50%	0.121
methods of detecting methylation	MSP	8	0.52(0.48;0.56)	0.82(0.79;0.84)	2.38(1.97;2.87)	0.61(0.53;0.70)	4.29(3.32;5.53)	0.7196	0	0.997
	qMSP	1	——	——	——	——	——	——	100	0
study design	perspective	2	0.36(0.29;0.42)	0.89(0.86;0.91)	3.22(2.39;4.36)	0.73(0.66;0.80)	4.46(3.02;6.58)	——	0	0.86
	Retrospective	7	0.62(0.57;0.66)	0.75(0.70;0.79)	2.25(1.69;3.00)	0.52(0.46;0.60)	4.46(3.05;6.53)	0.7036	14%	0.323
source of the control group	Healthy People	6	0.60(0.55;0.65)	0.81(0.75;0.85)	2.45(1.77;3.37)	0.53(0.46;0.61)	4.80(2.98;7.73)	0.6622	25.20%	0.245
	Non-cancerous stomach disease patients	3	0.43(0.37;0.49)	0.84(0.81;0.87)	2.56(1.61;4.07)	0.69(0.59;0.82)	4.27(3.04;5.99)	0.7114	0	0.884

### 3.6 Sensitivity analysis

**Figure 5:**
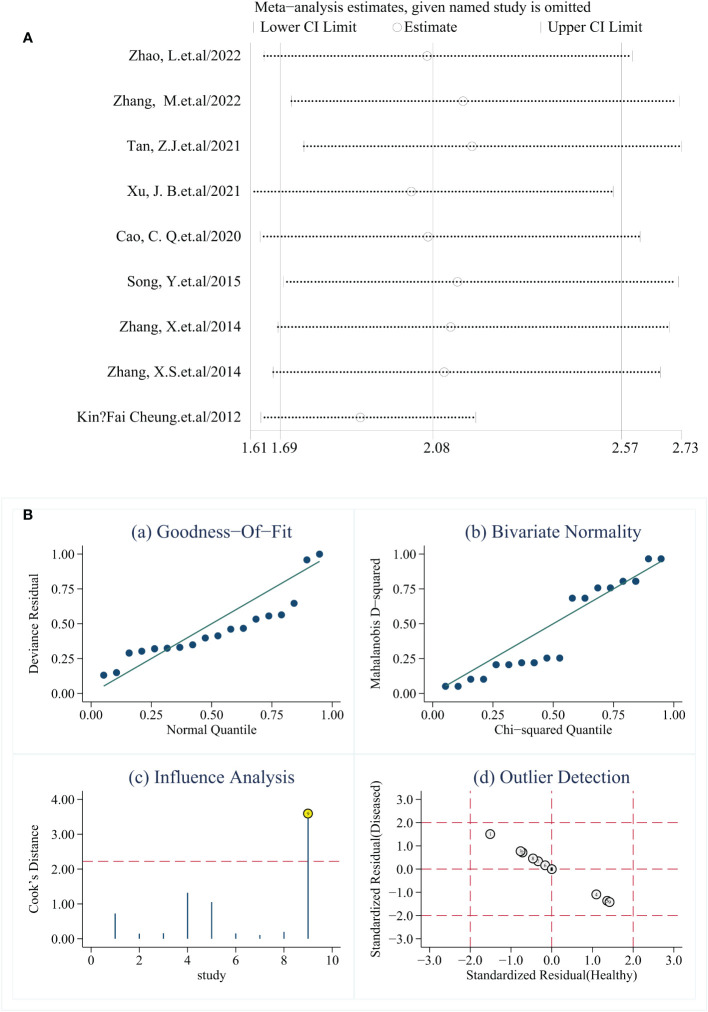
**(A)** Sensitivity analysis (one-by-one elimination method). **(B)** Sensitivity analysis. **(A, B)** show different presentations of the sensitivity analysis results. In **(B)**, the data in **(a, b)** are evenly distributed in a straight line, **(d)** no yellow literature appears, representing stable results, and the 9th literature in Figure **(c)** is marked yellow, suggesting that this literature affects the stability of the results of this study.

Sensitivity analysis was conducted by excluding individual studies one by one, and the results showed no significant changes in SEN, SPE, and DOR, indicating that the results were robust (**Figure 5**).

### 3.7 Publication bias

**Figure 6:**
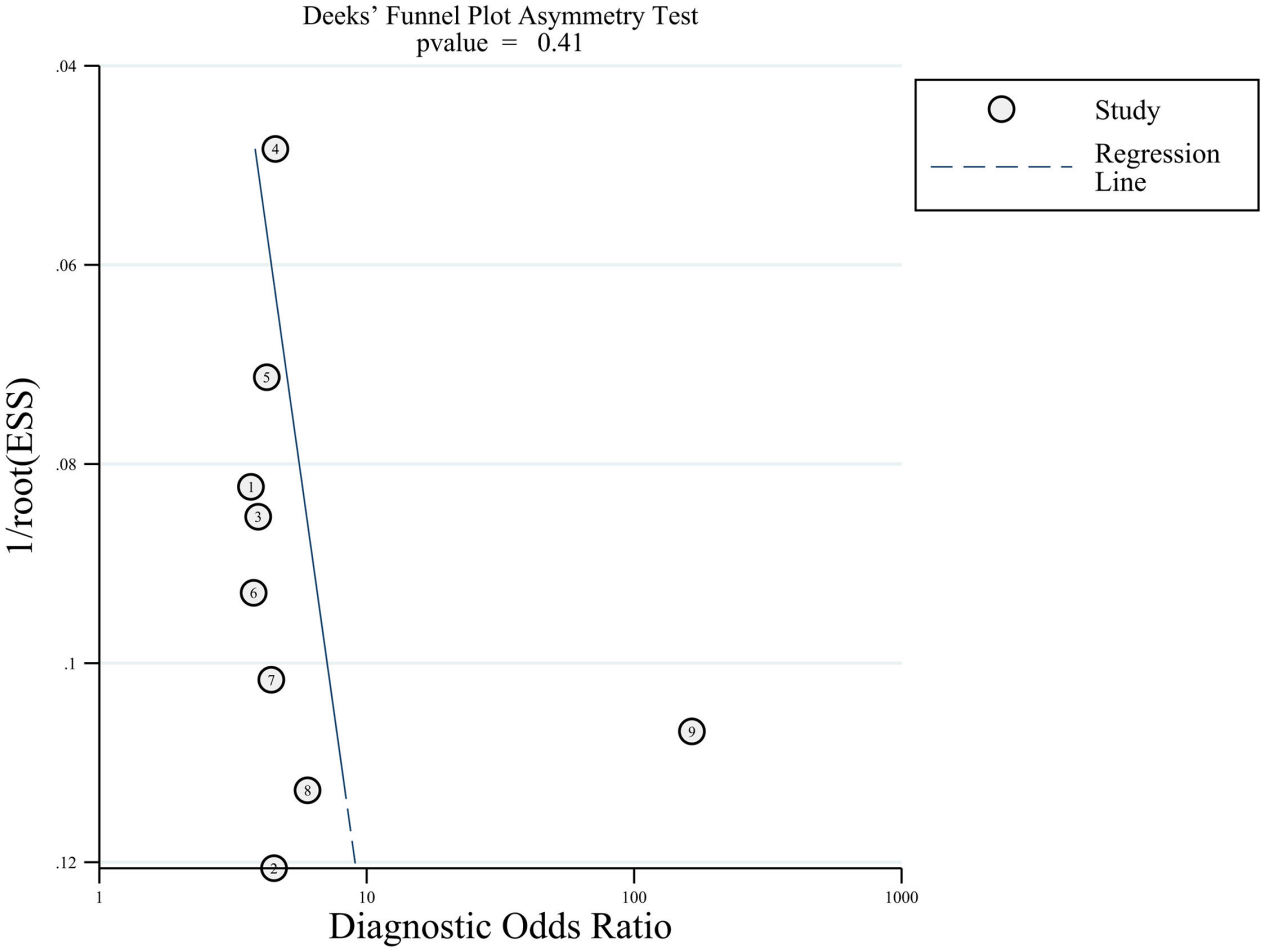
Deeks funnel plot of RNF180 gene methylation for GC diagnosis.

Deeks funnel plot was drawn to test publication bias, and the results showed that the distribution around each research point was symmetric (P=0.41), suggesting a small possibility of publication bias (**Figure 6**).

## 4 Discussion

Due to a lack of timely physical examinations and/or limitations in the level of early diagnosis, gastric cancer remains one of the major threats to human health. Although gastroscopic biopsy is currently an effective method for the early detection of gastric cancer, endoscopic clinicians must have a high level of operational and pathological experience to perform the test. Furthermore, the biopsy is difficult to detect subtle lesions, and there is a risk of internal bleeding and perforation due to irregular surgical procedures. To address these issues, an increasing number of diagnostic studies are focusing on the discovery of tumor biomarkers and the development of molecular biology instrumentation (30). Because blood biomarkers are noninvasive, inexpensive, and clinically feasible, they may be considered the screening method of choice for gastric cancer. Several studies are currently being conducted to investigate the diagnostic utility of various biomarkers for gastric cancer. An earlier meta-analysis of tumor markers in GC discovered that overall positivity rates for all these markers were low (CEA 24.0%, CA-199 27.0%, CA724 29.9%), and even lower in stage I GC (CEA 13.7%, CA199 9.0%, CA724 12.0%) (31). Although Yang et al. study’s found that the combined sensitivity of CA72-4, CEA, CA125, and CA19-9 could reach 66.0%, the sensitivity of individual biomarkers was lower, 33.0%, 25.5%, 31.1%, and 38.7%, respectively (32). The degree of methylation of specific genetic regions has been found to be directly related to lymph node metastasis, TNM stage, tumor pathological classification, and prognosis (33, 34). Given the ease of sample collection, the detection of DNA methylation in peripheral blood can be used as a molecular biological test for diagnosing disease and predicting patient prognosis, opening a new avenue for noninvasive cancer detection (35, 36). There is growing evidence that plasma RNF180 methylation is linked to gastric cancer, and RNF180 methylation has great potential as a diagnostic and prognostic tumor marker, with promising research prospects (16, 37). Due to the vulnerability of a single individual clinical study to small sample size, experimental technique, and experimental design (38). Therefore, this study investigated the diagnostic value of RNF180 methylation assay for gastric cancer by systematically evaluating all relevant clinical studies in the current field, and provided a reference for the application of tumor markers for the early diagnosis of gastric cancer.

The RNF180 methylation test had a specificity of 0.80 (95% CI: 0.72 - 0.87) in distinguishing gastric cancer patients from controls in our meta-analysis of 1531 participants from 9 research. However, its sensitivity was only 0.54 (95% CI: 0.45 - 0.62), which could be attributed to the confinement of lesions to localized areas and the insufficient amount of circulating free DNA that undergoes methylation in plasma, limiting its diagnostic value in the screening of gastric cancer (39). Despite its modest sensitivity, it has greater specificity than most plasma tumor markers. In fact, when compared to the sensitivity of various other tumor indicators, the RNF180 gene methylation assay performs admirably. That is, RNF180 gene methylation assays have the same sensitivity as other biomarkers but much higher specificity. Rutter et al. proposed the HSROC model as an extension of the fixed-effects comprehensive receiver operating characteristic curve model for the combined evaluation of sensitivity and specificity of several diagnostic studies (40). The results of the HSROC model showed a β of 0.31 (95% CI: -0.16, 0.78), *P*=0.194, indicating that the SROC was symmetric. In this study, Lambda, an effect index representing diagnostic test discriminatory capacity, was 1.38 (1.01, 1.75), indicating that the RNF180 gene methylation test has a relatively high diagnostic accuracy in gastric cancer diagnosis. For clinical decision-making, likelihood ratios and posterior probability are preferable. Because of the heterogeneity among studies, we conducted Meta-regression and subgroup analysis to explore the sources of heterogeneity. When subgroup analysis was performed on study design, method of detecting methylation, sample size, and source of control group, the within-group heterogeneity was still large, and the above factors could not explain the heterogeneity among studies well. Combined with the full text, the possible sources of heterogeneity were considered to be the different RNF180 methylation cutoff values among the included studies. However, some studies did not report the selection method of cutoff values and explain its rationality, resulting in the inability to further identify the sources of heterogeneity. Although the funnel plot showed a low likelihood of publication bias, the accuracy of the study results may have been compromised because the gray literature was not retrieved.

In conclusion, the results of this study showed that the plasma RNF180 gene methylation test is more realistic and reliable in assisting gastric cancer diagnosis, which fully demonstrated its important role in gastric cancer diagnosis. Therefore, the RNF180 gene methylation test is an effective predictive biomarker for gastric cancer. It can be used as a screening tool for gastric cancer, especially for subjects with poor physical conditions and unable to tolerate gastroscopy. This is the first systematic review and meta-analysis of plasma RNF180 gene methylation in gastric cancer diagnosis. The findings show that RNF180 gene methylation has a high specificity but a low sensitivity, which can lead to missed diagnoses and has limitations in clinical application and practice. To provide a better reference for clinical decision-making, clinical attention should be paid to the comprehensive analysis and judgment of plasma tumor markers, traditional imaging detection, and histopathological findings. Furthermore, the current clinical studies have a high quality of evidence, according to QUADAS-2, and thus the results of this study are reliable. Meanwhile, the consistency of the sensitivity analysis results, fully supported the reliability of our findings.

Several shortcomings of this study are worth considering: 1) All samples and related statistics are from China, and regional bias may exist, so the influence of ethnic and geographical factors on the selection bias of the study population cannot be excluded; 2) gray literature and conference abstracts were not retrieved, which may be led to publication bias; 3) The number of included studies was small and their quality was average, which to some extent reduced the credibility of the Meta-analysis results.

## 5 Conclusion

The discovery of promising biomarkers is critical for early detection and diagnosis of gastric cancer. The methylation status of RNF180 gene has diagnostic value for gastric cancer and has the potential to be used as a new tumor marker for the diagnosis of gastric cancer in clinical practice, according to our meta-analysis results based on nine studies. However, due to the current studies’ quality and quantity limitations, the conclusions drawn from a systematic review should therefore be interpreted cautiously, more high-quality studies are required in the future to further investigate the relationship between RNF180 gene methylation and gastric cancer.

## Data availability statement

The original contributions presented in the study are included in the article/**Supplementary Material**. Further inquiries can be directed to the corresponding author.

## Author contributions

TW contributed to the conception and design, acquired and interpreted the data, and critically revised the manuscript. YZ contributed to the conception, analysis, and drafting of the manuscript. JW contributed to the design, interpreted the data, and drafted the manuscript. YL critically revised the manuscript. All authors contributed to the article and approved the submitted version

## References

[1] Sung H Ferlay J Siegel RL Laversanne M Soerjomataram I Jemal A . Global cancer statistics 2020: Globocan estimates of incidence and mortality worldwide for 36 cancers in 185 countries. CA: Cancer J Clin (2021) 71(3):209–49. doi: 10.3322/caac.21492 33538338

[2] Cao CQ Chang L Wu Q . Circulating methylated septin 9 and ring finger protein 180 for noninvasive diagnosis of early gastric cancer. Transl Cancer Res (2020) 9(11):7012–21. doi: 10.21037/tcr-20-1330 PMC8799148 35117307

[3] Wang M Yang Y Xu J Bai W Ren X Wu H . Circrnas as biomarkers of cancer: A meta-analysis. BMC Cancer (2018) 18(1):303. doi: 10.1186/s12885-018-4213-0 29554887 PMC5859638

[4] Shimada H Noie T Ohashi M Oba K Takahashi Y . Clinical significance of serum tumor markers for gastric cancer: A systematic review of literature by the task force of the Japanese gastric cancer association. Gastric Cancer (2015) 17(1):26–33. doi: 10.1007/S10120-013-0259-5 23572188

[5] Necula L Matei L Dragu D Neagu AI Mambet C Nedeianu S . Recent advances in gastric cancer early diagnosis. World J Gastroenterol (2019) 25(17):2029–44. doi: 10.3748/wjg.v25.i17.2029 PMC6506585 31114131

[6] Moore LD Le T Fan G . DNA Methylation and its basic function. Neuropsychopharmacology (2013) 38(1):23–38. doi: 10.1038/npp.2012.112 22781841 PMC3521964

[7] Flanagan J Verma M . Cancer epigenetics: Risk assessment, diagnosis, treatment, and prognosis. New York: Springer (2015) pp. 5–63. doi: 10.1007/978-1-4939-1804-1 25568925

[8] Sun J Zheng M-Y Li Y-W Zhang S-W . Structure and function of septin 9 and its role in human malignant tumors. World J Gastrointestinal Oncol (2020) 12(6):619. doi: 10.4251/wjgo.v12.i6.619 PMC7340996 32699577

[9] Mikeska T Craig JM . DNA Methylation biomarkers: Cancer and beyond. Genes (2014) 5(3):821–64. doi: 10.3390/genes5030821 PMC4198933 25229548

[10] Ogawa M Mizugishi K Ishiguro A Koyabu Y Imai Y Takahashi R . Rines/Rnf180, a novel ring finger gene-encoded product, is a membrane-bound ubiquitin ligase. Genes to Cells (2008) 13(4):397–409. doi: 10.1111/j.1365-2443.2008.01169.x 18363970

[11] Cheung KF Lam CN Wu K Ng EK Chong WW Cheng AS . Characterization of the gene structure, functional significance, and clinical application of Rnf180, a novel gene in gastric cancer. Cancer (2012) 118(4):947–59. doi: 10.1002/cncr.26189 21717426

[12] Zhao B Tsai YC Jin B Wang B Wang Y Zhou H . Protein engineering in the ubiquitin system: Tools for discovery and beyond. Pharmacol Rev (2020) 72(2):380–413. doi: 10.1124/pr.118.015651 32107274 PMC7047443

[13] Han F Sun LP Liu S Xu Q Liang QY Zhang Z . Promoter methylation of Rnf180 is associated with H.Pylori infection and serves as a marker for gastric cancer and atrophic gastritis. Oncotarget (2016) 7(17):24800–9. doi: 10.18632/oncotarget.8523 PMC5029743 27050149

[14] Wei F Ba S Jin M Ci R Wang X E F . Rnf180 inhibits proliferation and promotes apoptosis of colorectal cancer through ubiquitination of Wisp1. Front Cell Dev Biol (2021) 8:623455. doi: 10.3389/fcell.2020.623455 33553163 PMC7862563

[15] Liu H Yang P Li X Jia Y . Ring finger protein 180 is associated with biological behavior and prognosis in patients with Non−Small cell lung cancer. Oncol Lett (2020) 20(4):1–. doi: 10.3892/ol.2020.11898 PMC7412726 32802159

[16] Wu Z Liu H Sun W Du Y He W Guo S . Rnf180 mediates Stat3 activity by regulating the expression of rhoc *Via* the proteasomal pathway in gastric cancer cells. Cell Death Dis (2020) 11(10):1–11. doi: 10.21203/rs.3.rs-21786/v1 33082325 PMC7575565

[17] Sun W Ma G Zhang L Wang P Zhang N Wu Z . Dnmt3a-mediated silence in Adamts9 expression is restored by Rnf180 to inhibit viability and motility in gastric cancer cells. Cell Death Dis (2021) 12(5):1–12. doi: 10.1038/s41419-021-03628-5 33931579 PMC8087691

[18] Deng J Guo J Guo X Hou Y Xie X Sun C . Mediation of the malignant biological characteristics of gastric cancer cells by the methylated cpg islands in Rnf180 DNA promoter. Oncotarget (2016) 7(28):43461. doi: 10.18632/oncotarget.9494 27223257 PMC5190037

[19] Deng J Liang H Zhang R Hou Y Liu Y Ying G . Clinical and experimental role of ring finger protein 180 on lymph node metastasis and survival in gastric cancer. J Br Surg (2016) 103(4):407–16. doi: 10.1002/bjs.10066 26805552

[20] Deng J Liang H Ying G Zhang R Wang B Yu J . Methylation of cpg sites in Rnf180 DNA promoter prediction poor survival of gastric cancer. Oncotarget (2014) 5(10):3173. doi: 10.18632/oncotarget.1888 24833402 PMC4102801

[21] Han F Liu S Jing J Li H Yuan Y Sun LP . Identification of high-frequency methylation sites in Rnf180 promoter region affecting expression and their relationship with prognosis of gastric cancer. Cancer Manag Res (2020) 12:3389–99. doi: 10.2147/cmar.S246995 PMC7231750 32494203

[22] Zhang X Zhang X Sun B Lu H Wang D Yuan X . Detection of aberrant promoter methylation of Rnf180, Dapk1 and Sfrp2 in plasma DNA of patients with gastric cancer. Oncol Lett (2014) 8(4):1745–50. doi: 10.3892/ol.2014.2410 PMC4156173 25202403

[23] Whiting PF Rutjes AW Westwood ME Mallett S Deeks JJ Reitsma JB . Quadas-2: A revised tool for the quality assessment of diagnostic accuracy studies. Ann Intern Med (2011) 155(8):529–36. doi: 10.7326/0003-4819-155-8-201110180-00009 22007046

[24] Zhao L Liu Y Zhang S Li M . Plasma methylated Rnf180 for noninvasive diagnosis of gastric cancer. BioMed Res Int (2022) 2022:6548945. doi: 10.1155/2022/6548945 36246966 PMC9556199

[25] Zhang M Ding C Zhang K Kong LY . Methylation of plasma Pax5 and Rnf180 genes in patients with gastric cancer and its clinical significance. J Clin Exp Med (2022) 21(12):1269–72. doi: 10.3969/j.issn.1671-4695.2022.12.010

[26] Tan Z Zhao YG Xiao XY . Methylations and clinical significances of plasma Pax5 and Rnf180 genes in patients with gastric cancer. Chin J Pract Diagnosis Ther (2021) 35(6):584–7. doi: 10.13507/j.issn.1674-3474.2021.06.010. (in Chinese).

[27] Xu JB Song JL Wang TM Zhu WC Zuo LY Wu JZ . A combination of methylation and protein markers is capable of detecting gastric cancer detection by combined markers. Epigenomics (2021) 13(19):1557–70. doi: 10.2217/epi-2021-0080 34632818

[28] Song Y Zhang X Sun B Zhang X Huang S Huang Z . Value of combined detection of plasma Sfrp2, Rnf180 gene methylation in clinical diagnosis of gastric cancer. Chin J Gastroenterol (2015) 20(1):19–23. doi: 10.3969/j.issn.1008-7125.2015.01.006

[29] ZX S Zhang X Sun B Song Y Lu H Wang D . Diagnostic value of promoter methylation and protein expression of plasma Rnf180 gene in gastric cancer. Chin J Clin Oncol (2014) 41(22):1432–6. doi: 10.3969/j.issn.1000-8179.20141067

[30] Hanahan D Weinberg RA . Hallmarks of cancer: The next generation. cell (2011) 144(5):646–74. doi: 10.1016/j.cell.2011.02.013 21376230

[31] Shimada H Noie T Ohashi M Oba K Takahashi Y . Clinical significance of serum tumor markers for gastric cancer: A systematic review of literature by the task force of the Japanese gastric cancer association. Gastric Cancer (2014) 17(1):26–33. doi: 10.1007/S10120-013-0259-5 23572188

[32] Yang A-P Liu J Lei H-Y Zhang Q-W Zhao L Yang G-H . Ca72-4 combined with cea, Ca125 and Ca19-9 improves the sensitivity for the early diagnosis of gastric cancer. Clinica chimica Acta (2014) 437:183–6. doi: 10.1016/j.cca.2014.07.034 25086284

[33] Wang N Sui F Ma J Su X Liu J Yao D . Site-specific hypermethylation of Runx3 predicts poor prognosis in gastric cancer. Arch Med Res (2016) 47(4):285–92. doi: 10.1016/j.arcmed.2016.07.011 27664488

[34] Tahara T Arisawa T . DNA Methylation as a molecular biomarker in gastric cancer. Epigenomics (2015) 7(3):475–86. doi: 10.2217/epi.15.4 26077432

[35] Gómez-Zamudio JH Mendoza-Zubieta V Ferreira-Hermosillo A Molina-Ayala MA Valladares-Sálgado A Suárez-Sánchez F . High thyroid-stimulating hormone levels increase proinflammatory and cardiovascular markers in patients with extreme obesity. Arch Med Res (2016) 47(6):476–82. doi: 10.1016/j.arcmed.2017.03.017 27986128

[36] Avincsal MO Jimbo N Fujikura K Shinomiya H Otsuki N Morimoto K . Epigenetic down-regulation of sox 2 is an independent poor prognostic factor for hypopharyngeal cancers. Histopathology (2018) 72(5):826–37. doi: 10.1111/his.13436 29143365

[37] Han F Liu S Jing J Li H Yuan Y Sun L-P . Identification of high-frequency methylation sites in Rnf180 promoter region affecting expression and their relationship with prognosis of gastric cancer. Cancer Manage Res (2020) 12:3389. doi: 10.2147/CMAR.S246995 PMC7231750 32494203

[38] Moher D Hopewell S Schulz KF Montori V Gøtzsche PC Devereaux PJ . Consort 2010 explanation and elaboration: Updated guidelines for reporting parallel group randomised trials. Int J Surg (2012) 10(1):28–55. doi: 10.1016/j.jclinepi.2010.03.004 22036893

[39] Zhao Q-T Guo T Wang H-E Zhang X-P Zhang H Wang Z-K . Diagnostic value of Shox2 DNA methylation in lung cancer: A meta-analysis. OncoTargets Ther (2015) 8:3433—9. doi: 10.2147/ott.s94300 PMC4657794 26640383

[40] Rutter CM Gatsonis CA . A hierarchical regression approach to meta-analysis of diagnostic test accuracy evaluations. Statistics in Medicine (2001) 20(19):2865–84. doi: 10.1002/sim.942. 11568945

