# Supplementary material for: Diagnostic value of plasma RNF180 gene methylation for gastric cancer: A systematic review and meta-analysis
Source: Front Oncol. 2023 Jan 10;12:1095101. doi: 10.3389/fonc.2022.1095101 (PMC9872154; doi:10.3389/fonc.2022.1095101)
Supplement: Supplementary file 1 [file DataSheet_1.docx]

**Supplementary Material**

**1. Search keywords for the systematic review:**

• methylation

• rnf180; rines; ring finger protein 180

• Stomach Neoplasm; gastric cancer; gastric carcinoma; stomach cancer; Stomach Neoplasm; stomach carcinoma; gastric adenocarcinoma;

• gastric; stomach

• cancer; carcinoma; tumor; malignancy; neoplasm; adenocarcinoma

**2. Search formula**

**2.1 PubMed: 11 articles**

(((("Stomach Neoplasms"[Mesh]) OR ((((((gastric cancer[Title/Abstract]) OR (gastric carcinoma[Title/Abstract])) OR (stomach cancer[Title/Abstract])) OR (Stomach Neoplasm[Title/Abstract])) OR (stomach carcinoma[Title/Abstract])) OR (gastric adenocarcinoma[Title/Abstract]))) OR (((gastric[Title/Abstract]) OR (stomach[Title/Abstract])) AND ((((((cancer[Title/Abstract]) OR (carcinoma[Title/Abstract])) OR (tumor[Title/Abstract])) OR (malignancy[Title/Abstract])) OR (neoplasm[Title/Abstract])) OR (adenocarcinoma[Title/Abstract])))) AND ((((rnf180[Title/Abstract]) OR (rines[Title/Abstract])) OR (ring finger protein 180[Title/Abstract])) OR ("RNF180 protein, human" [Supplementary Concept]))) AND ((methylation[Title/Abstract]) OR ("DNA Methylation"[Mesh]))

**2.2 Web of science: 12 articles**

(((((TS= (gastric cancer)) OR TS= (gastric carcinoma)) OR TS= (stomach cancer)) OR TS= (stomach carcinoma)) OR TS= (Stomach Neoplasms)) OR TS= (gastric adenocarcinoma) AND TS= (RINES OR "ring finger protein 180 " OR RNF180) AND TS=(methylation)

**2.3 Embase: 15 articles**

#13 #6 AND #9 AND #12

#12 #10 OR #11

#11 methylation:ti,ab,kw

#10 'methylation'/exp

#9 #7OR #8

#8 'ring finger protein 180' :ti,ab,kw OR rines:ti,ab,kw OR rnf180:ti,ab,kw

#7 'ring finger protein 180'/exp

#6 #1 OR #4 OR #5

#5 'stomach neoplams' :ti,ab,kw OR 'gsastric cancer' :ti,ab,kw OR ' gastric carcinoma' :ti,ab,kw OR 'stomach cancer' :ti,ab,kw OR 'stomach noeoplasm' :ti,ab,kw OR 'stomach carcinoma' :ti,ab,kw OR ' gastrie adenocarcinoma':ti,ab,kw

#4 #2 AND #3

#3 cancer:ti,ab,kw OR carcinoma:ti,ab,kw OR tumor:ti,ab,kw OR malignancy :ti,ab,kw CR neoplasm:ti,ab,kw CP adenocarcinoma:ti,ab,kw

#2 stomach:ti,ab,kw OR gastric:ti,ab,kw

#1 'stomach cancer'/exp

**2.4 The Cochrane library: 0 articles**

#1 MeSH descriptor: [Stomach Neoplasms] explode all trees

#2 (stomach): ti,ab,kw OR (gastric):ti,ab,kw

#3 (cancer*): ti,ab,kw OR (carcinoma*): ti,ab,kw OR (neoplas*): ti,ab,kw OR (tumor*): ti,ab,kw OR (lymphoma*): ti,ab,kw

#4 #2 AND #3

#5 (Gastric Neoplasms): ti,ab,kw OR (stomach neoplasms) : ti,ab,kw OR(Gastic Cancer) : ti,ab,kw OR(stomach Cancer) : ti,ab,kw OR(gastric carcinom:) : ti,ab,kw

#6 #1 OR #4 OR #5

#7 (rnf180): ti,ab,kw OR (rines) : ti,ab,kw OR ("ring finger protein 180"): ti,ab,kw

#8 (methylation): ti,ab,kw

#9 #7AND #6 AND #8

**2.5 CNKI****: 21 articles**

(主题:胃癌〔精确) ) OR (主题;胃瘤〔精确)) OR (主题:胃肿物(精确) ) AND ((主题: rnf180(精确)) OR (主题: Rines(精确)) OR (主题: ring finger protein 180(精确))) AND ((主题:甲基化(精确)))

**2.6 CBM: 8 articles**

("RINES"[常用字段:智能]OR "RNF180"[常用字段:智能]) AND ("胃癌"[常用字段:智能] OR "胃瘤"[常用字段:智能] OR "胃肿物"[常用字段:智能]) AND ("甲基化"[不加权:扩展])

**2.7 WanFang Data: 19 articles**

主题:(胃癌 OR 胃瘤 OR 胃肿物) and 主题:(rnf180 OR rines OR ring finger protein 180) and 主题:(甲基化)

**2.8 VIP: 9 articles**

(((M=胃癌 OR M=胃瘤) OR M=胃肿物) ) AND (((M=rnf180 OR M=Rines) OR M=ring finger protein 180)) AND (M=甲基化)
